# Analysis of the Functionality of the Feed Chain in Olive Pitting, Slicing and Stuffing Machines by IoT, Computer Vision and Neural Network Diagnosis

**DOI:** 10.3390/s20051541

**Published:** 2020-03-10

**Authors:** Alberto Lucas Pascual, Antonio Madueño Luna, Manuel de Jódar Lázaro, José Miguel Molina Martínez, Antonio Ruiz Canales, José Miguel Madueño Luna, Meritxell Justicia Segovia

**Affiliations:** 1 Food Engineering Department, Technical University of Cartagena, 30203 Cartagena, Spain; info@albertolucas.es (A.L.P.); josem.molina@upct.es (J.M.M.M.); 2 Aerospace Engineering and Fluid Mechanical Department, University of Seville, 41013 Seville, Spain; antonio@madueno.es; 3 Engineering Department, Miguel Hernández University of Elche, 03312 Orihuela, Spain; acanales@umh.es (A.R.C.); meritxelljusticia@gmail.com (M.J.S.); 4 Graphics Engineering Department, University of Seville, 41013 Seville, Spain; jmadueno@us.es

**Keywords:** Internet of things (IoT), table olive pitting, slicing and stuffing machines, artificial neural networks (ANNs), CM1K chip, Intel Curie chip, Teensy

## Abstract

Olive pitting, slicing and stuffing machines (DRR in Spanish) are characterized by the fact that their optimal functioning is based on appropriate adjustments. Traditional systems are not completely reliable because their minimum error rate is 1–2%, which can result in fruit loss, since the pitting process is not infallible, and food safety issues can arise. Such minimum errors are impossible to remove through mechanical adjustments. In order to achieve this objective, an innovative solution must be provided in order to remove errors at operating speed rates over 2500 olives/min. This work analyzes the appropriate placement of olives in the pockets of the feed chain by using the following items: (1) An IoT System to control the DRR machine and the data analysis. (2) A computer vision system with an external shot camera and a LED lighting system, which takes a picture of every pocket passing in front of the camera. (3) A chip with a neural network for classification that, once trained, classifies between four possible pocket cases: empty, normal, incorrectly de-stoned olives at any angles (also known as a “boat”), and an anomalous case (foreign elements such as leafs, small branches or stones, two olives or small parts of olives in the same pocket). The main objective of this paper is to illustrate how with the use of a system based on IoT and a physical chip (NeuroMem CM1K, General Vision Inc.) with neural networks for sorting purposes, it is possible to optimize the functionality of this type of machine by remotely analyzing the data obtained. The use of classifying hardware allows it to work at the nominal operating speed for these machines. This would be limited if other classifying techniques based on software were used.

## 1. Introduction

Olives were pitted and stuffed by hand until the 1970s. As labor costs gradually increased, the need to mechanize both processes arose. The industrialization of the olive industry progressed at a slow pace, and the first continuous olive pitting machines appeared in 1975 [1]. Current models have improved and are able to pit olives at a rate up to 2500 olives/min. However, there has been a lack of electronic improvements because the ongoing mechanical improvements in olive pitting machines have limited the incorporation of new technologies and fine adjustments. It is worth mentioning however some of the few advances in this area, including performance optimization and remote error detection [2]. The olive pitting minimum error rate of traditional systems is 1–2% (if the machine is correctly adjusted) and cannot be improved through mechanical adjustments although this error can increase due to the poor calibration of an olive pitting machine. The consequences of these error increases include a greater fruit loss and food safety issues, because the pitting process is not completely ensured, it may result in a low-quality product. The growing competitiveness in producer countries, due to a globalized market, and the increase of food safety measures have led to new technological solutions. These solutions are aimed at improving the reliability of olive pitting machines during the pitting process by removing the stone completely and increasing their productivity for better competition with other producers.

As a biological product characterized by a heterogeneous morphology, olives challenge detection technologies. Therefore, neural networks are the tools chosen to identify every possible case. Cases using deep learning and computer vision have been confirmed to deliver great results and are promising solutions for solving the aforementioned problem. Yunchao et al. [3] presented an article of interest on the use of real-time detection in recycled aggregate concrete technology. However, for this particular case, it does not prioritize the processing speed. Therefore, this technology does not have the same applicability in the present article. Another study that could be considered for determining an applicable solution is the analysis developed by Mingyou et al. [4], who used a multi-camera system, which is interesting for the analysis of several pitting machines or increase the perception range of vision. However, the use of several cameras is not the subject of this study. Therefore, it is necessary to explore other solutions that provide sufficient processing speed to analyze images at the speed prescribed by the pitting machines. Although these studies use interesting technology for the detection and classification of images, their results are not applicable here because processing speed is not relevant.

There are applications which use computer visions with rapid detection technologies for industrial purposes like product classification or image analysis. These systems use morphological operations to extract the sought characteristics in the image through filters and specific analysis algorithms like Canny Edge, Otsu, local binary pattern algorithm, K-means, etc. In order to develop these algorithms, specific software is used, such as the integrated development setting of Microsoft Visual Studio and specific computer vision libraries, as in the case with Opencv. Maoyon, et al. [5] proposes a study using these types of techniques in the classification of apples on an industrial level. Although the described procedures are of great interest, these types of techniques require high-performance computing hardware and elevated costs for the type of application evaluated in the current analysis, where more economic solutions and reduced hardware size are sought. Lucas et al. [6] propose a specific example done with olives which uses these fast detection techniques based on C++ programming language and OpenCV artificial vision libraries.

Specific studies in the agri-food sector and the related behavior of neural networks have been considered. It is worth mentioning the studies conducted by Guichao et al. [7,8,9] on detection systems for the artificial visualization of fruits through RGB space color analysis and robotic systems. In the case of the classification of apples, Yang et al. [10] provide positive results. Yang’s investigation used three layers of 9-6-3 neurons, with 96.6% accuracy. Nagata et al. [11] developed a grading system for fruit and vegetables using neural network technologies, obtaining a high percentage of accuracy for strawberries and green peppers (94% to 98% and 89%, respectively). Likewise, Behrooz et al. [12] applied machine vision and artificial neural network (ANN) for modelling and controlling the grape drying process, offering a new method for predictive modelling of the grape drying process for the on-line monitoring and controlling of the process.

There have been some techniques used to classify table olives using computer vision and neural networks. Gatika et al. [13] proposed an interesting paper about olive fruit recognition using neural networks. Olive fruit recognition is performed by analyzing RGB images taken from olive trees. Another study used a neural network based on the backpropagation method. Mancuso et al. [14] identified olive (*Olea europaea*) cultivars using artificial neural networks. Backpropagation neural networks (BPNNs) were used to distinguish 10 olive (*Olea europaea* L.) cultivars that originate throughout the Mediterranean basin. The book *Computer Vision Technology for Food Quality Evaluation*, 2nd Edition [15], includes a specific chapter based on the different technologies use to analyze the quality of food. Chapters 11 to 13 describe the quality evaluations of apples, citrus fruits, and strawberries, respectively. Of particular interest to the problem at hand, Chapter 14 [16] explains the classification and evaluation of table olives and describes how to classify them by color, shape, or external defects made by insects. The usual way to classify olives is by using computer vision. That paper analyzes the images captured by a camera connected to a PC; these images allow the analysis of 66 olives per matrix. In that paper, the author used a Bayesian math model for pre-classification to perform this process. Neural network software was used, with 15 sorting parameters and a hidden layer. The result was successful. The network was able to classify more than four types of olives. The results, however, could be improved by using high-resolution images.

Previous examples employed software-based neural networks, where the processing speed is not relevant. Thus, although the detection and classification technology in these studies is applicable, it is necessary to look for technology that achieves high processing speeds. In addition, it is preferable to use systems with limited physical implementation that can be installed with low intrusion at lower costs, instead of traditional hardware, such as industrial PC’s. Therefore, neural network technology based on a physical chipset ensures its successful implementation and low cost.

The possible applications of this type of chip are very broad. The following article is interesting because CM1K neural chip of General Vision Inc. (Petaluma, CA 94952 USA) was implemented in a common industrial process used to fill bottles in factories. This process is quite simple; however, it may be necessary to use an intelligent device to inspect the process and ensure food security [17]. In another example, parallel neural network chips were used offshore for fish inspection before filleting [18]. Each network chip system uses four neural network chips (accounting for 312 neurons) based on a natively parallel, hard-wired architecture that performs real-time learning and nonlinear classification (RBF). The use of CM1K in real time verifies its use for the purposes of the present study.

The combination of computer vision and neural networks offers a way to perform tasks that could be more complex. Liu et al. [19] proposed a neural network chip for license plate recognition. This chip combines a video image-processing module with a neural network module by using equalized image processing algorithms and network classification algorithms. Santu Sardar et al. [20] published a paper based on automated facial recognition, which is a technique employed in a wide range of practical applications, including personnel access control and identification systems. Image recognition is simpler than image processing methods for facial recognition, mainly due to the lack of a fixed pattern for comparison purposes; these applications reveal the tremendous possibilities of CM1K.

Later CM1K chipsets have delivered satisfactory results. An interesting aspect to consider in future research would be to comparison our chosen chip with these other technologies described in the following studies [21,22,23,24,25,26,27,28,29,30].

The use of the IoT is widely extended nowadays and there are numerous cases, for example [31,32,33,34,35] in precision agriculture, irrigation, temperature control, monitoring of the agricultural production process or automated-olive-chain. In the case presented in this paper, we are going to be able to monitor and analyze the operation of DRR machines through the internet.

## 2. Materials and Methods

In this section, the internal structure of the CM1K chip (Section 2.1 and Section 2.2) and the implementation of Matlab or neural networks with characteristics similar to those of the CM1K chip are analyzed (Section 2.3).

A chip such as the CM1K has a limitation in that the entry vector it can support is reduced in this case (256 bytes), meaning that if it is going to process an image, that image cannot be larger in pixel size than this value (256 bytes) and that it could only be B/W with 256 levels of gray. In order to assure that a neural network with these characteristics (equipped with high speed as it is pure hardware, but of limited vector entry) can be acceptable for the purpose of classification in olive pitting machines, a neural network is simulated in Matlab which works with a delay and allows us to assure that correct classification will be carried out in various categories with images of just 16 × 16 pixels in B/W.

In Section 2.4 and Section 2.5 the physical implementation of the classification system is shown with a real olive pitting machine and in Section 2.6 the commercial version of the CM1K chip that has been used in the trials is analyzed. For these sections, the description supplied in [36] will be used by the authors of this paper.

In Section 2.7 the communication hardware of the CM1K-PC for classification in real time is described and lastly, in Section 2.8, the IoT system to control the olive pitting, slicing and stuffing machine (DRR in Spanish) and data analysis is analyzed.

### 2.1. Neural Network

In this work a supervised neural network with backpropagation has been chosen because there are no relevant differences with respect to the calculation processes used by other networks. Moreover, this system is one of the most widespread systems used for image identification purposes, and it is widely used in the MATLAB Neural Network Toolbox employed in this paper. Backpropagation is the most efficient way to set this value [37]. First, errors are calculated in output units by considering the difference between the desired and predetermined values. Next, they propagate through the network using the weights and obtain the minimum value in the most optimal way.

The software emulation of a neural network is computationally demanding, which indicates that its operations will take a long period of time to be carried out. The use of a chip that physically implements a neural network (as in this paper with the CM1K chip or Intel Curie chip) will speed up the training and response processes of the neural network. A comparison with other existing hardware neural networks like Google Coral Edge TPU [38], Intel^®^ Movidius™ Neural Computer Stick 2 [39] or Nvidia-Jetson-Nano [40] and optimized results will be provided in future papers.

### 2.2. Operation of the CM1K Chip

The CM1K is a pattern recognition accelerator chip that is trainable in real-time by learning examples [41]. It is a fully parallel silicon neural network for either learning or recognition [42], that can store and process information simultaneously. It is composed of four modules [43], has two possible classifiers: K-nearest neighbor (KNN) or radial basis function (RBF) [44] and uses the “Winner-Takes-All” strategy [45]. It has 1024 neurons of 256 bytes. Intel Curie chip is a diminished version with only 128 neurons of 128 bytes.

### 2.3. The MATLAB Neural Network Toolbox

In this work we are using the MATLAB Neural Network [46] by using a specific network called ‘autoencoder’ for classification purposes [47,48], it will allow us to analyze the effect of image resolution on the sorting capacity of the network.

#### 2.3.1. Preliminary Tests: Maximum Resolution Available

The maximum resolution implemented under camera board is 144 × 176 meeting the size requirements of the pocket according to used lenses. After the first test using the above-mentioned resolution, a 25,344 pixels at 1-byte color depth grayscale image is obtained, which implies a high processing rate (nearly two minutes using a 2.5 GHz CPU, RAM 8 GB, Microsoft Windows 10, Redmond, Washington, USA, and MathWorks MATLAB 2019, Massachusetts, USA).

Preliminary tests suggest that processing optimization measures need to be carried out. These measures will focus on two main aspects:

- Establishing a region of interest (ROI) on the image.
- Testing different resolutions that allow the identification of the image at the minimum processing rate.

In addition to these aspects, it is important to consider the processing rate of the physical chips: Intel Curie (128 neurons with a 128-byte input vector) and NeuroMem CM1K (1024 neurons with a 256-byte input vector).

Considering the above-mentioned restrictions, the maximum resolution of the image must be between 128 and 256 pixels for a square image, as finally used: 16x16 pixels for NeuroMem CM1K 256-byte chip and 11 × 11 pixels (121 bytes < 128 bytes) for Intel Curie chip.

#### 2.3.2. Preliminary Tests: Minimum Resolution

Several tests to identify the minimum resolution accepted by physical chips are carried out with the purpose of setting the lowest processing rate. To do so, real images are to be processed in MATLAB and it should be estimated if the system is able to identify them.

The first step is to set the ROI, which must be a part of the image with enough information in order to identify the position of the olive in the pocket. Therefore, the olive must be completely displayed on the image.

**Figure 1:**
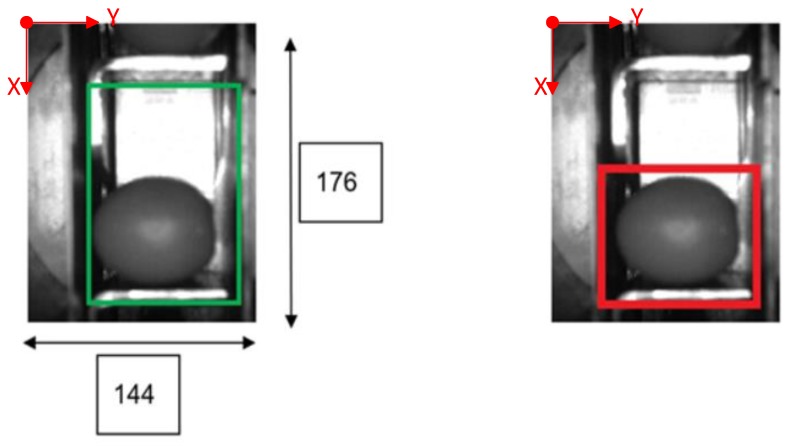
Example of the use of region of interest (ROI) in an olive in a normal position. The red point corresponds to the origin of coordinates for pixels. The green rectangle comprises the pocket within its walls and depth and limited by the previous and the next pocket of the feed chain. The red rectangle is the ROI.

**Figure 2:**
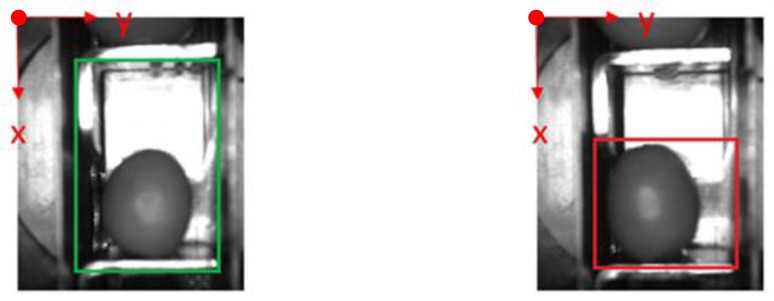
Example of the use of ROI in an olive in a “boat” position between [80°, 90°] and [−80°, −90°].

The following Figure 1 and Figure 2 show some examples of 176 × 144 pixel-images with the (X,Y) reference system. As shown in the figures, the olive is restricted in the *X*-axis (the same pocket confines the olive to its space) and, therefore, the selection of the parameters of ROI in that axis is constant. There is a possibility for the olive to move in *Y*-axis, consequently leading to a deviation of the dimensions of ROI. However, it is empirically proven that, due to the inclination of the chain and the upward movement of the olive, gravity, inertia and some elements of the machine (brushes, for example) forces the olive to stay in a similar position. Variations may be identified in orientation, which is the subject of this paper.

**Figure 3:**
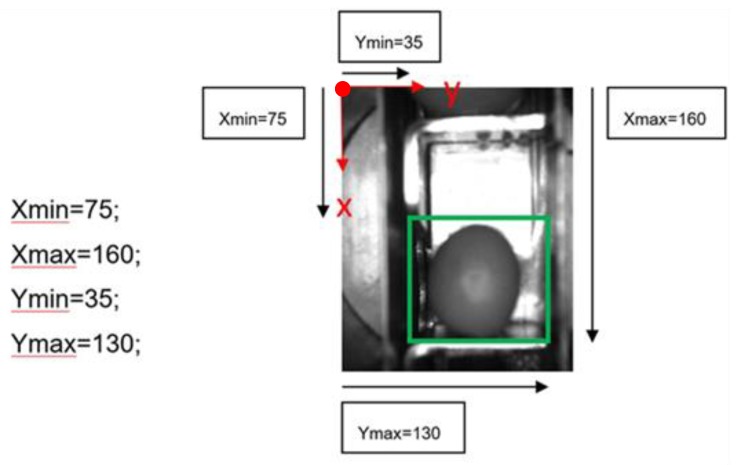
Selected ROI.

**Figure 4:**
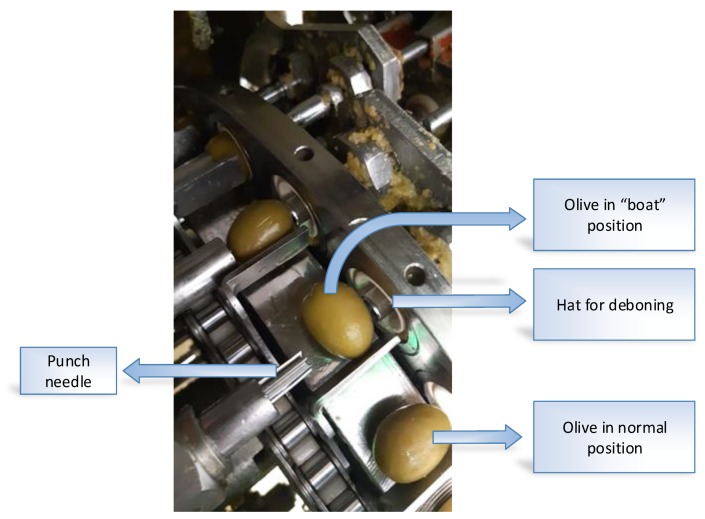
Punch needles in an olive pitting machine.

After previous considerations, the following conditions are set out (measured in pixels and referred to the origin of coordinates, Figure 3):

Once the ROI parameters in X and Y are set, several tests are carried out in order to establish if the system is still able to identify images using the selected resolutions. A set of 10,000 images of 176 × 144 pixels in B/W of the olives in normal position and boat and empty buckets are available, there are no anomalous cases (i.e., leaves, small branches, stones, double olives, small parts) in this set. This set is available to other researchers [49]. With an application in Matlab, each image is binarized and the orientation of each olive is calculated. For the tests described in this work, they are randomly selected in groups of 300 olives. The orientation of each olive (normal, intermediate position or boat) is defined by the position relative to the punches (Figure 4).

The classification by angles is as follows (see Table 1):

- [0°, 10°] and [0°, −10°] (Normal)
- [10°, 20°] and [−10°, −20°] (Intermediate)
- [20°, −30°] and [−20°, −30°] (Intermediate)
- [30°, −40°] and [−30°, −40°] (Intermediate)
- [40°, −50°] and [−40°, −50°] (Intermediate)
- [50°, −60°] and [−50°, −60°] (Intermediate)
- [60°, −70°] and [−60°, −70°] (Intermediate)
- [70°, −80°] and [−70°, −80°] (Intermediate)
- [80°, −90°] and [−80°, −90°] (Boat)

### 2.4. Hardware Used in Image Capture

The system developed has two functions:

- Obtain images for deferred analysis with Matlab, the Intel Curie and CM1K neural chips to evaluate the operation of the latter.
- Characterize the real-time operation through IoT of the pitting machines of an olive factory that reach speeds of up to 2500 olives/min.

**Figure 5:**
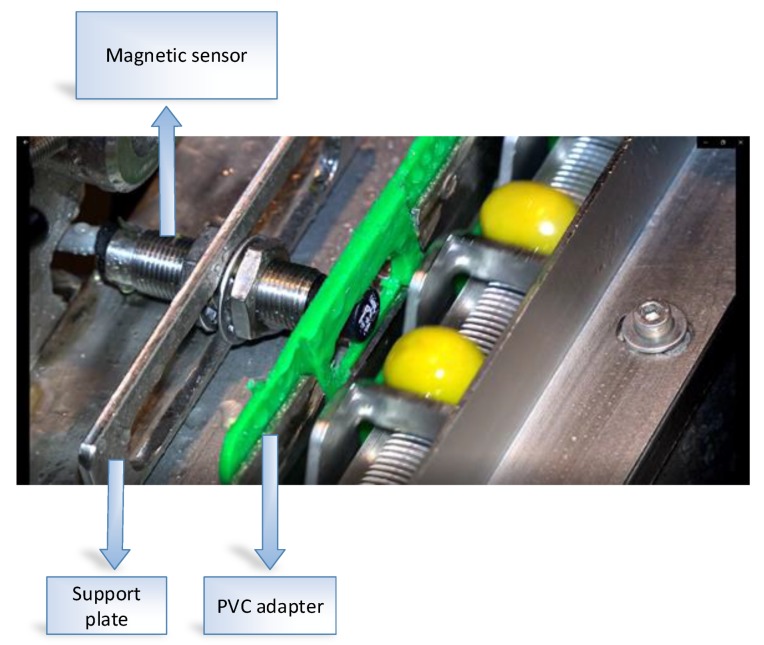
Magnetic sensor used to detect the passage of the pockets in the chain.

**Figure 6:**
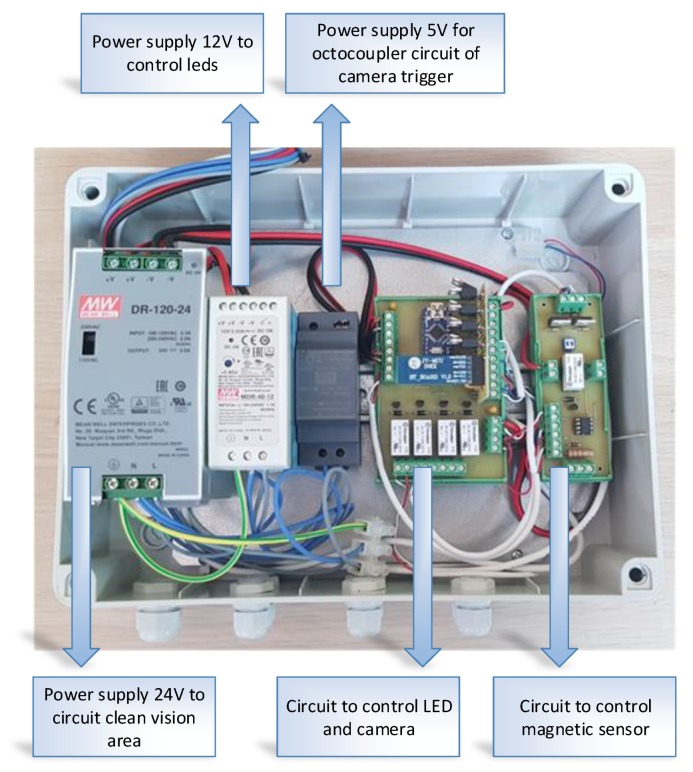
Electronic circuit of the external trigger and LED.

**Figure 7:**
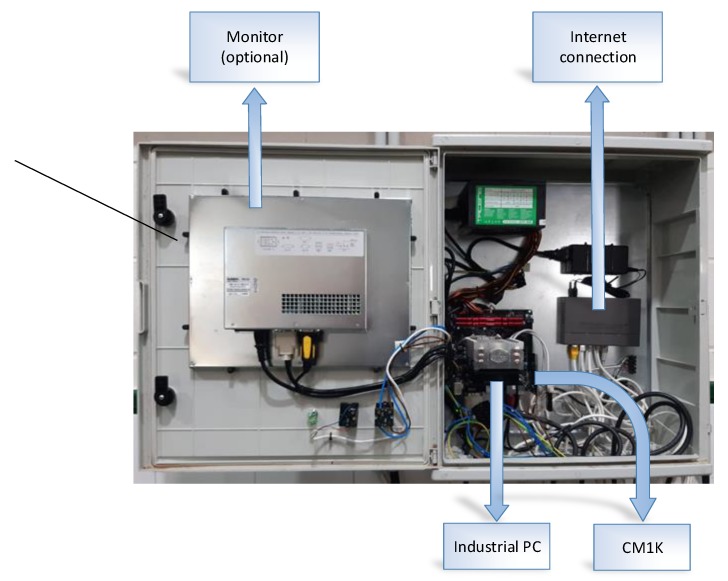
Electronic circuit of the PC with CM1K chip.

The system consists of two parts:

- The hardware that lets the camera [50] control and lighting based on a magnetic sensor that allows to obtain images of each bucket facing the camera (Figure 5 and Figure 6).
- A personal computer (PC) which allows, for the first case, the storage of images for deferred analysis and in the second case together with a CM1K chip for real-time analysis of the same over the internet with an application developed in Qt Creator [51] (Figure 7).

### 2.5. Application for IoT Management of DRR Machines

**Figure 8:**
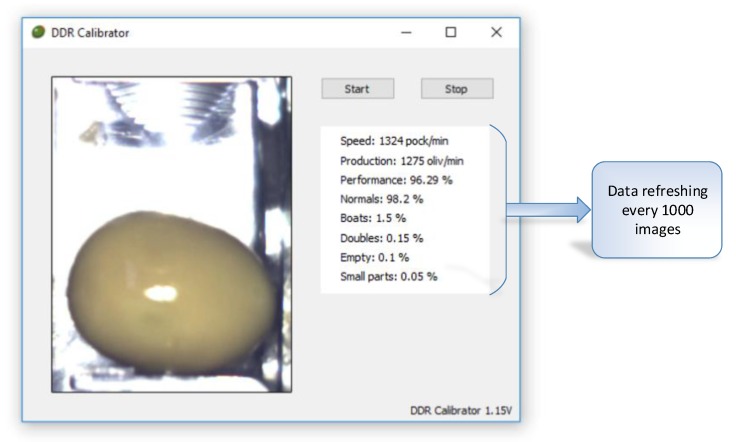
Qt Creator application for IoT management of olive pitting, slicing and stuffing (DRR) machines.

As we said, for the remote control of the DRR machines, an application in Qt Creator (Figure 8) has been developed which allows the most important operating parameters to be seen every 1000 images. This data is sent via Dropbox [52] to a folder which can later be consulted to analyze the operation of the machine (see Section 3.3).

**Figure 9:**
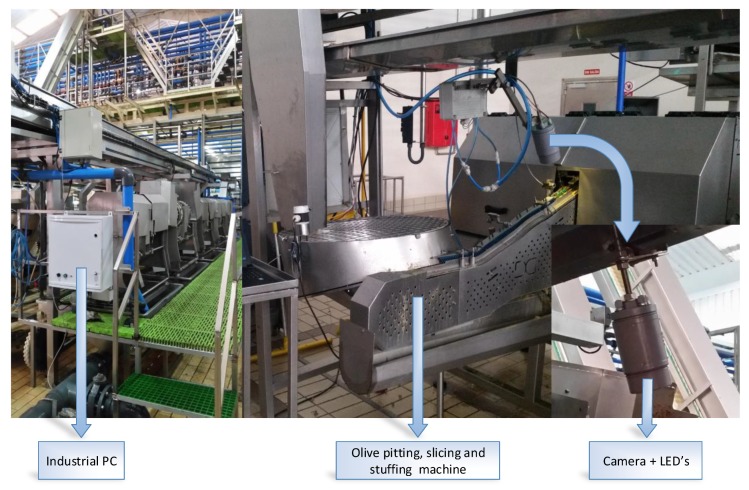
The system implemented on a “DRR machine”.

The application previously determines the size in pixels of the olive to generate two cases not contemplated by the neuronal network: small parts and doubles, these two cases are not sent to the neural network. Figure 9 shows the system (Industrial PC, “DRR machine”, camera and led) in a table olive factory.

### 2.6. Neural Chips Used

As mentioned before in Section 2.2., two neuromorphic chips have been used in this work. The first one is an Intel Curie from an Arduino-Genuino 101, which includes an ANN module (128 neurons with a 128-byte input vector). This module is a scale case of the second chip CogniMem CM1K with 1024 neurons and an input vector of 256 bytes, and it has been used in a BrainCard [53,54]. Both chips use RBF or k-NN techniques for sorting purposes.

121 pixels images (11 × 11) will be processed with the Intel Curie Arduino 101 and with the CM1K chip. 256 pixels images (16 × 16) only with the CM1K chip. We have used three classification options:

- First, there is a complex and thorough process on both chips by which olives are classified according to existing angles, see previous Section 2.3.2.
- Later, an intermediate position is set to simplify the rank of angles and the following positions are only taken into account: normal (interval [0°, −10°] and [0°, 10°]), “boat” ([−80°, −90°] and [80°, 90°]), intermediate (from 10° to 80° and −10° to −80°) and empty pocket.
- Finally, this classification is simplified even more using only three out of four pocket cases: normal, “boat” and empty.

For training purposes, a maximum of 300 olives per case has been taken into account as an empirical value to prevent overtraining, which may lead the system to detect even the most trifling detail and, therefore, an unnecessary overuse of neurons. Every case has been tested using 300 random olives replicated 10 times, which accounts for 3000 per every case.

### 2.7. CM1K-PC Hardware Communication for Real Time Classification

**Figure 10:**
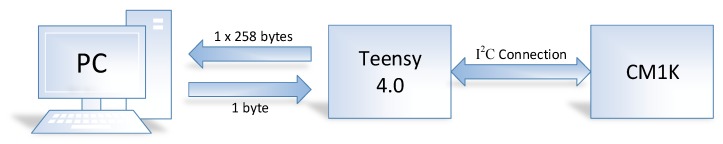
PC-CM1K communication system.

One of the main problems found in the implementation of this neural chip for use in olive pitting machines, which reach speeds of up to 2500 olives/min, is the speed of communication between the chip and the external PC supplying the image to be classified. The problem arises because this chip uses I^2^C [55] communication and requires an intermediate interface (a UART-USB microcontroller with a FT232RL [56] or a CH340G [57] converter) which transforms the I^2^C communication into serial. Normally, this communication reaches a maximum speed of 115,200 bauds with the PC, Figure 10. Conversely, if the information is transmitted in 1 byte packets (default case), the communication is slowed because the PC-microcontroller combination has to restart the process of serial communication every time 1 byte is sent.

To eliminate this problem, a microcontroller has been used which directly includes direct conversion to USB generating a “virtual port”. Specifically, these two options have been analyzed:

(1) The INTEL CURIE [58] (which internally incorporates a limited version of the CM1K chip with 128 neurons with a 128 byte vector) but which also can be used just for a part of the communication with the PC At the present time, it is no longer produced.
(2) A TEENSY 4.0 [59] which includes a ARM Cortex-M7 (NXP iMXRT1062) operating at a speed of 600 MHz.

Conversely, to avoid restarting the communication series for each byte, complete 256 byte packets have been sent with each image (16 × 16 bytes) which allow them to arrive to the CM1K in just one delivery. Each delivery, is made up of 258 bytes: 1 byte as the header (to indicate the type of instruction, for example training the neural network or challenging), 256 bytes of data (with a 16 × 16 image) and a last byte, in case the of a challenge, includes an answer from the neural network (olive in a normal position, boat position, empty, anomaly, etc.).

In Table 2, the differences in the times expressed in ms during the communication with a UART-USB, an INTEL CURIE and a TEENSY 4.0 standard converter are shown. The average value is of 1000 packages of 258 × 1 byte in the first row and in the second a single package of 258 bytes.

If we consider that the reception process for the information of the byte classified has an unappreciable duration, we find that the delivery of 1 × 258 byte in the case of CH340G, CURIE and TEENSY show processing rates of 2.72, 3.87 and 6.56 images per second respectively or 163.2, 232.2 and 393.6 olives/min, clearly insufficient for the standard speed of the olive pitting machines which can reach 2500 olives/min.

In the case of the delivery of 258 bytes with a CH304 chip, it would reach the level of 44.6 images per second (2677.3 olives/min), but we consider that any load process in the PC could generate a loss of processed images, and for that reason we will only use the cases of delivery of a single data package of 258 bytes with CURIE or TEENSY.

### 2.8. IoT System to Control the DRR Machine and Data Analysis

**Figure 11:**
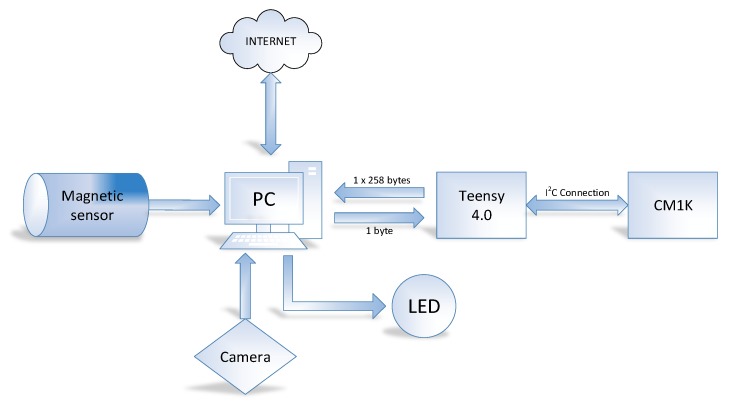
IoT control system.

An IoT system has been implemented for the analysis and remote control consistent with a client connected to an olive pitting machine which includes: a PC with Windows 10 and specific hardware which has an air conditioning system, the magnetic sensor, camera, LED lighting and chip CM1K (Figure 11).

The system is connected to the internet and every 1000 images generates information via an application (see Section 2.5) which is stored in a file and is sent by Dropbox, so that it can be consulted at any time from any other PC Another specific application has been developed to allow the analysis of this data and with it, the evolution of the operation of the olive pitting machine (see Section 3.3). In order to control the system remotely, a remote desktop from Google [60] is used.

## 3. Results

### 3.1. Results Obtained Using a MATLAB Neural Network

The structure of the autoencoder of the neural network has been trained with 45 iterations, fixed by the MATLAB neural network library. Three settings have been tested: The first one uses 1-byte depth greyscale images, 10 × 10 pixels ROI-scaled, the second one uses 1-byte depth greyscale images, 11 × 11 pixels ROI-scaled and the last one uses 1-byte depth greyscale images, 16 × 16 pixels ROI-scaled.

**Figure 12:**
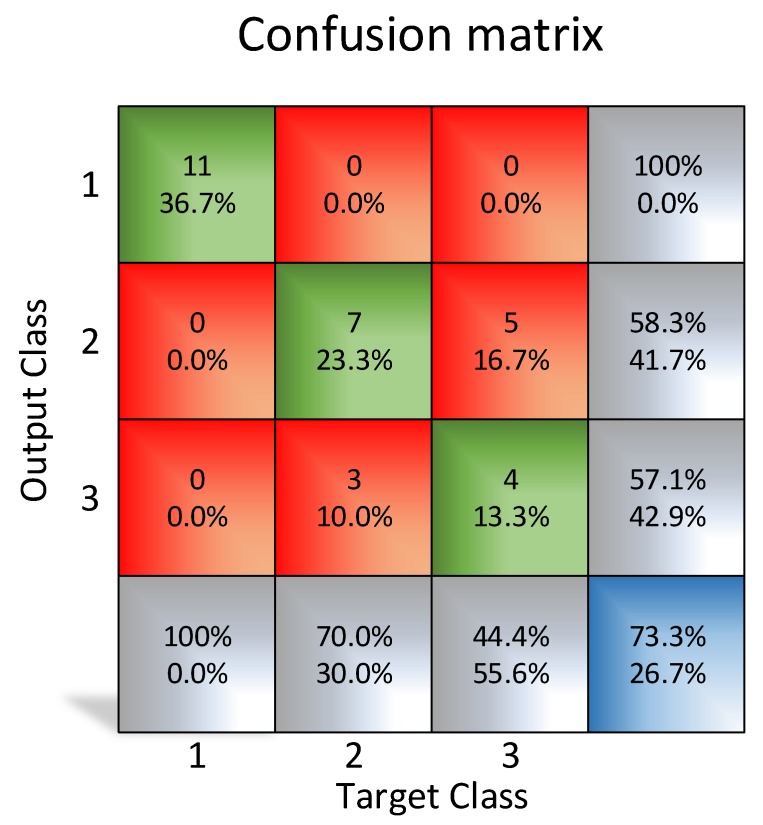
Confusion matrix using 10 × 10 resolution images.

For training purposes, a set of 9 images of empty pockets, 11 of “boat” olives and 10 of normal olives has been used. Unusual case images were not included since the purpose of the test was to prove the feasibility of the classification of these low-resolution images using a neural network. The results using a resolution of 10x10 pixels show an unsatisfactory outcome, as shown in Figure 12 with 26.7% error rate.

**Figure 13:**
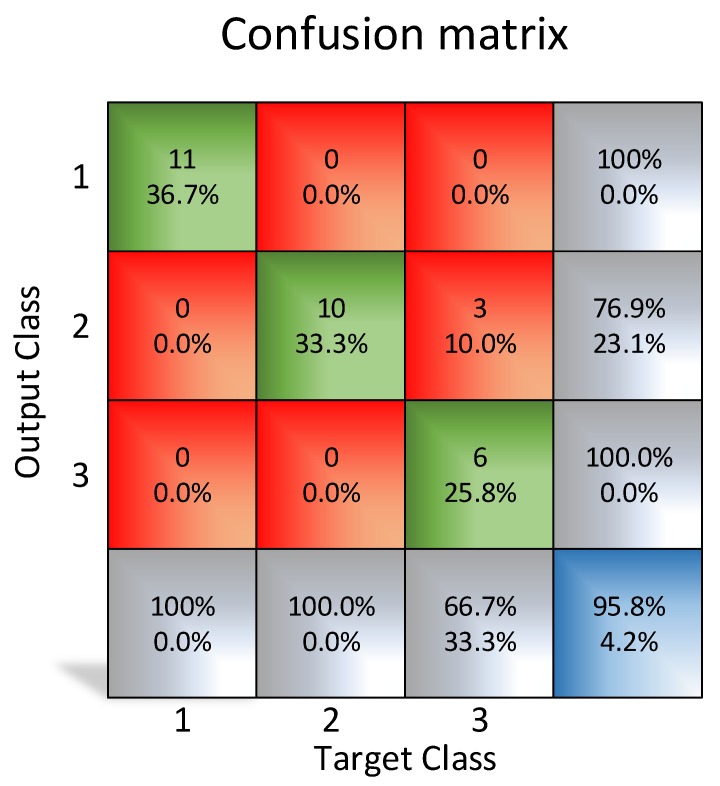
Matrix of the obtained results using MATLAB (11 × 11 pixels resolution).

**Figure 14:**
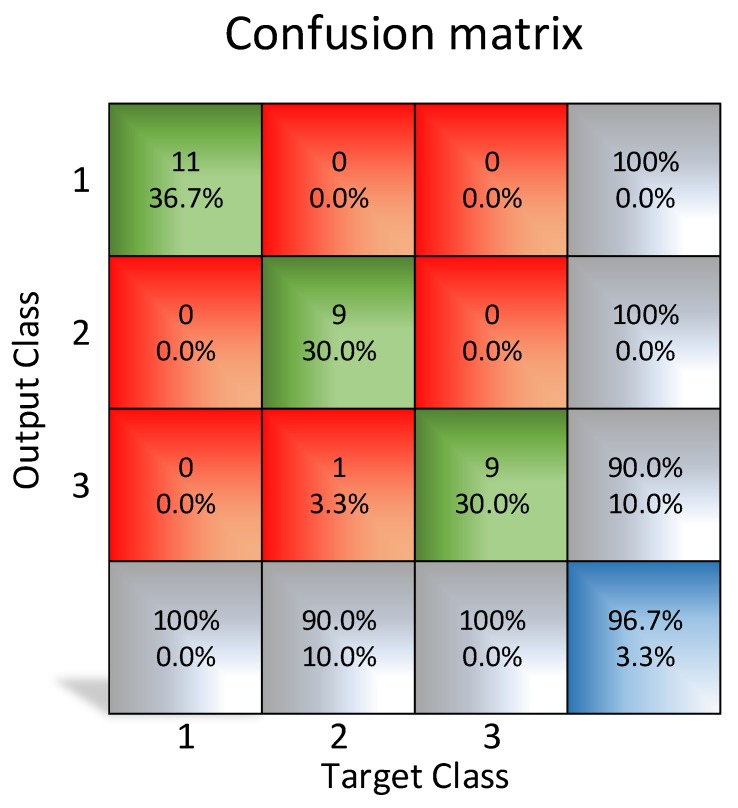
Matrix of the obtained results using MATLAB (16 × 16 pixels resolution).

As shown in Figure 13, the neural network has carried out an appropriate classification (<5% error rate) with 11 × 11 pixels images processed. Tests have been repeated with 16 × 16 pixels images (Figure 14), and the results were better (<4% error rate), which means that it is also possible to classify the cases with the images (11 × 11 pixels).

### 3.2. Results Obtained Using Neuromorphic Chips

The following results are given (Table 3 and Table 4) according to the chip that has been used, the type of classification (degrees, intermediate position and simple) and number of neurons. The total error rate is the average obtained of the ten repetitions.

The optimal option is Braincard 256 chip (0.63% error rate < 1%) and only needs four neurons to classify between normal, empty and boat olives. If we use an Intel curie chip error rate is 4.80% < 5% and only needs four neurons too. This means that the suggested system is capable of detecting up to a 95–99% of the deficiencies (boats and empty) presented by the machine.

### 3.3. Analysis of the Results of the Operation of the DRR Machine

**Figure 15:**
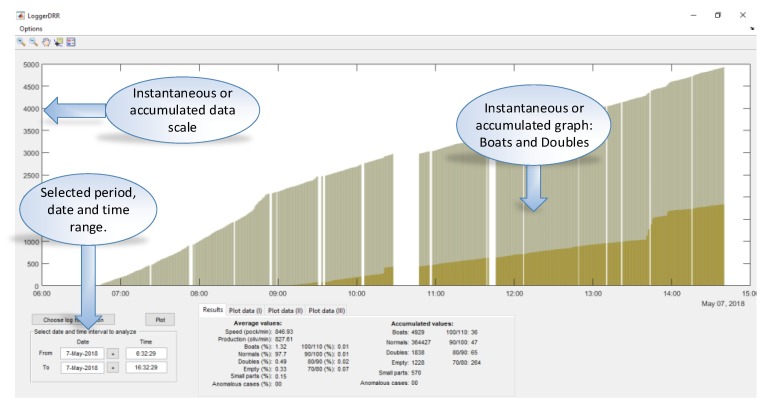
Graphical User Interface (GUI) created in Matlab to analyze data from the “DRR machines”.

**Figure 16:**
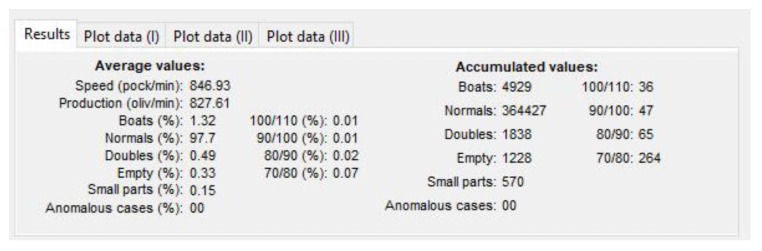
Settings dialogue box of the GUI from Matlab.

An application (“LoggerDRR”) has been developed to analyze the operation of the olive pitting machine with GUI by Matlab (Figure 15). The most relevant parameters of this application will be indicated below:

The representative data is indicated in the following box (Figure 16:

- Speed: Indicated in pockets per minute
- Production: Real value in olives per minute (without empty pocket)
- “Boat” olives, normal, double olives, empty, small pieces, anomalies: Percentage of the total production.
- Accumulated values: Data added in the selected period.

**Figure 17:**
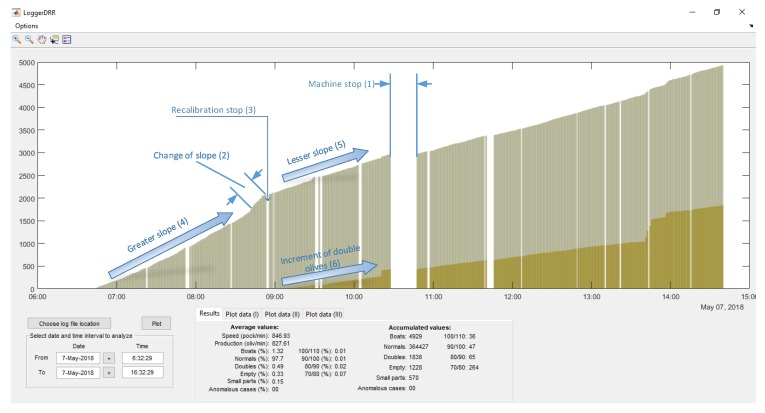
GUI of “boat” olives and doubles for the “DRR machine” diagnosis.

In Figure 17, a practical example of use is shown. There are two accumulated graphs, the olive green one corresponding to “boat” olives detected and the ochre color corresponding to double olives entering in the pocket. Each data update point corresponds to a count of 1000 olives.

The spaces correspond to periods when the machine was stopped (1), this can be due to changes in the feed chain (change of container where the olives come from) or stops to adjust the machine or personnel breaks.

In (2), the machine is observed to have a misalignment (due to the misalignment of the brush which straightens the olives in the feeding chain), followed by a stop for recalibration (3). Immediately after that, we can observe how the slope decreases (4)→(5) which means the number of “boat” olives has decreased. Additionally, the number of double olives (various in the same pocket) increases (6), meaning that the recalibration has not been optimal and the feed plate has been poorly adjusted.

**Figure 18:**
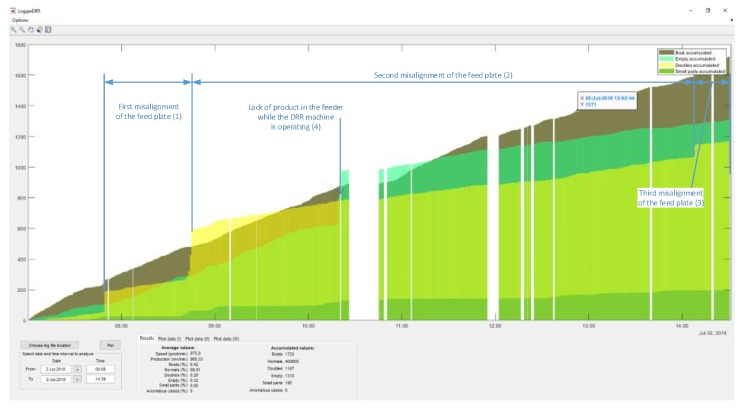
Multiple accumulated values (boats, empty, doubles and small parts).

Figure 18 shows an example of various combined parameters (boat accumulates, empty accumulated, doubles accumulated and small parts accumulated).

**Figure 19:**
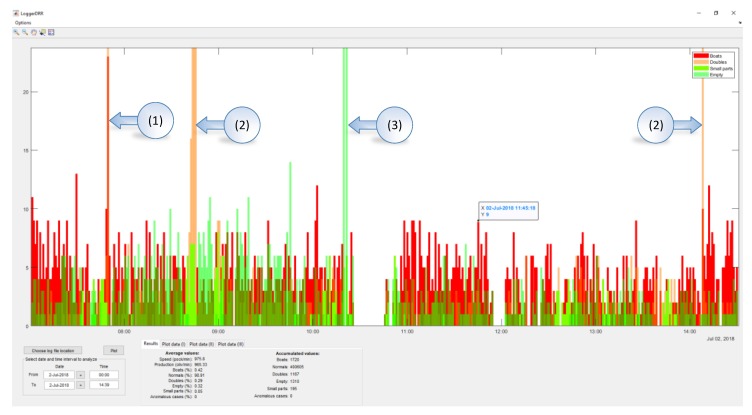
Instantaneous values for boats, doubles, small parts and empty.

This type of graph allows us to observe if the calibration process entails a comprehensive improvement in performance or, conversely, whether an improvement in a parameter after an adjustment entails a reduction in goodness of fit in another. In (1), (2) and (3) it can be observed how the feeding plate goes out of adjustment a little more each time, which has an impact on the number of doubles and small parts. During all of this time, the growth in the number of “boat” olives is monotonous which means that there are no misalignments in the straightening brush. For its part, the number of empty (4) increases abruptly each time a lack of product in the feeder when the DRR machine is working. At times, the data analysis without accumulating data can be interesting in order to see the effect of specific actions. In Figure 19, the peaks in the “boat” olives/doubles (1), of only doubles (2), empty (3) are observed due to the action of isolated adjustments or operation of the machine. In all of the cases shown, these actions are unfavorable (either calibration or operation).

**Figure 20:**
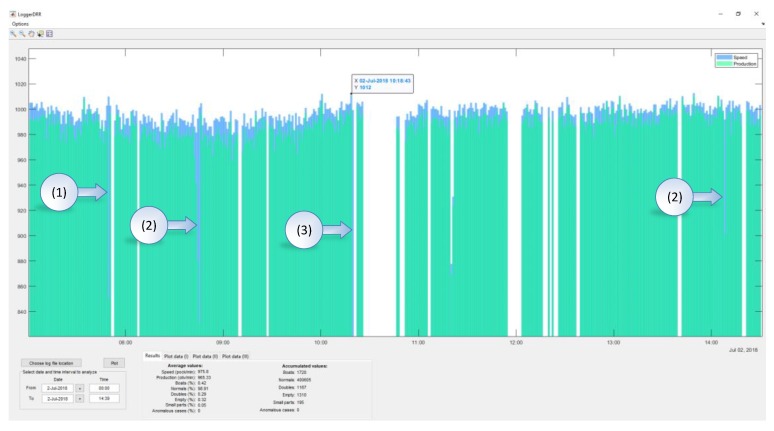
Instantaneous values of speed and production.

Finally, Figure 20 shows instances in which the performance decreases when working with a machine with a poorly adjusted feeding plate and straightening brush (1), only with the feed plate misaligned (2) or due to the lack of product in the feeder (3).

## 4. Conclusions

With the current paper, an IoT control system for a DRR machine has been developed which allows its operation to be improved:

An IoT system has been developed which allows for the reception of useful operational information for the DRR machine remotely in order to do an operational diagnosis based on this information, and, in this case, carry out calibration operations. This IoT system allows for configuration of the equipment via a remote desktop.

In the second place, the classification system has been made to work in an industrial setting at a nominal speed for the DRR machine (up to 2500 olives per minute) through the use of a PC-CM1K connection which uses a microcontroller with an Intel Curie or Teensy “virtual port” as an interface.

In the third place, the training and testing of a neural network based on a physical chip to classify olives in the feed chain of a pitting machine has been successfully completed. It has been proven that the use of Intel Curie or CM1K chips are suitable for simple sorting (normal, empty, boats), with error rates of less than 5% with the Intel Curie chip and less than 1% with the CM1K chip.

An application developed in Qt Creator has been used that previously detects for its study the cases with small parts of olives or double olives present in the pocket and separates them from those that are sent to the neural chip that correspond to normal, empty pockets and boats.

One limitation of the system currently developed is that it only allows for the diagnosis of the DRR machine so that the operator can recalibrate it. In a future paper, the possibility of ejecting those olives which are badly positioned (“boat” olives or doubles) or which have defects (small pieces) in the feeding chain will be presented. In this way, “boat” olives or doubles being badly de-stoned will be avoided. Likewise, it will assure that the olives which break upon entering in the feed chain causing small pieces do not get to the de-stoning area, thus avoiding, on the one hand, the loss of product (1–2%), and on the other hand, the accelerated deterioration of the punch needles and hats for deboning.

## Figures and Tables

**Table 1:** Images (both original and binarized) classified according to angular intervals.

**ANGLE [0°, 10º]**	**ANGLE [0°,−10°]**	**ANGLE [10°,20°]**	**ANGLE [−10°,−20°]**
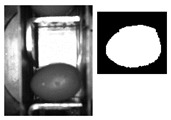	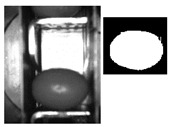	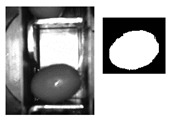	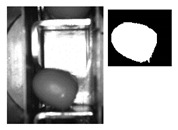
**ANGLE [20°,30°]**	**ANGLE [−20°,−30°]**	**ANGLE [30°,40°]**	**ANGLE [-30°,-40°]**
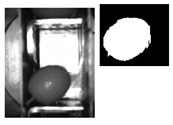	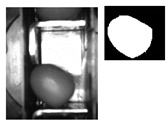	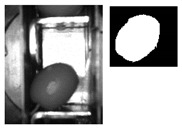	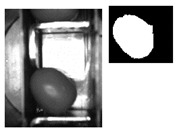
**ANGLE [40°,50°]**	**ANGLE [−40°,−50°]**	**ANGLE [50°,60°]**	**ANGLE [−50°,−60°]**
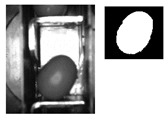	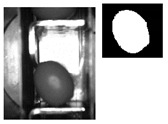	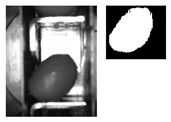	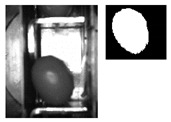
**ANGLE [60°,70°]**	**ANGLE [−60°,−70°]**	**ANGLE [70°,80°]**	**ANGLE [−70°,−80°]**
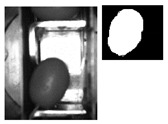	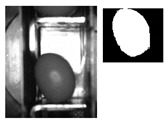	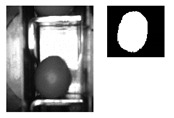	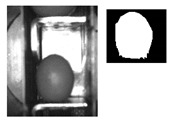
**ANGLE [80°,90°] or [−80°,−90°]**			
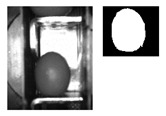			

**Table 2:** Average communication times in ms during the delivery of a 16 × 16 bytes image to be classified.

	CH340G	CURIE	TEENSY
1 BYTE	366.48	258.08	152.26
258 BYTES	22.41	5.04	0.78

**Table 3:** Results of the training patterns using a resolution of 11 × 11 pixels.

Type of Test		Degrees		Intermediate Position		Simple	
		Neuron	96	Neurons	24	Neurons	4
Board	Vector	Repeatability	Error	Repeatability	Error	Repeatability	Error
**Intel** **Curie**	**121**	1	19%	1	7%	1	6%
		2	15%	2	11%	2	9%
		3	7%	3	7%	3	4%
		4	11%	4	10%	4	6%
		5	13%	5	9%	5	6%
		6	14%	6	10%	6	6%
		7	8%	7	6%	7	3%
		8	7%	8	9%	8	2%
		9	12%	9	10%	9	3%
		10	7%	10	7%	10	3%
**Totals**			11.30%		8.47%		4.80%

**Table 4:** Results of the training patterns using a resolution of 1 6 × 16 pixels.

Type of Test		Degrees		Intermediate Position		Simple	
		Neuron	66	Neurons	24	Neurons	4
Board	Vector	Repeatability	Error	Repeatability	Error	Repeatability	Error
**Braincard** **(CM1K)**	**256**	1	8%	1	3%	1	1%
		2	9%	2	4%	2	0%
		3	10%	3	4%	3	0%
		4	12%	4	3%	4	2%
		5	13%	5	4%	5	1%
		6	13%	6	5%	6	1%
		7	15%	7	2%	7	0%
		8	16%	8	5%	8	1%
		9	16%	9	8%	9	1%
		10	13%	10	2%	10	0%
**Totals**			12.43%		4%		0.63%

## References

[1] Santos F.J. (1999). Siles New technologies in table olive processing. Grasas Aceites.

[2] Madueño A., Lineros M., Madueño J. System and Procedure Based on a Synchronism Sensor for the Detection of Malfunctions in Pitting Machines Olive and Filling Machines, Quantification and Optimization of Performance, Signaling, Monitoring and Remote Control. ES2529816A2. https://patents.google.com/patent/ES2529816A2/en.

[3] Tang Y., Li L., Wang C., Chen M., Feng W., Zou X., Huang K. (2019). Real-time detection of surface deformation and strain in recycled aggregate concrete-filled steel tubular columns via four-ocular vision. Robot. Comput. Integr. Manuf..

[4] Chen M., Tan Y., Zou X., Huang K., Li L., He Y. (2019). High-accuracy multi-camera reconstruction enhanced by adaptive point cloud correction algorithm. Opt. Lasers Eng..

[5] Nie M., Zhao Q., Xu Y., Shen T. Machine Vision-based Apple External Quality Grading. Proceedings of the Chinese Control and Decision Conference.

[6] Lucas A., Madueño A., De Jódar M., Molina J., Ruiz A. Characterization of the percentage of poorly positioned olives in pitting, rolling and filling machines for table olives (DRR). Proceedings of the X Congresso Ibérico de Agroengenharia.

[7] Lin G., Tang Y., Zou X., Li J., Xiong J. (2019). In-field citrus detection and localisation based on RGB-D image analysis. Biosyst. Eng..

[8] Lin G., Tang Y., Zou X., ·Xiong J., Fang Y. (2020). Color‑, depth‑, and shape‑based 3D fruit detection. Prec. Agric..

[9] Lin G., Tang Y., Zou X., Xiong J., Li J. (2019). Guava. Detection and Pose Estimation Using a Low-Cost RGB-D Sensor in the Field. Sensors.

[10] Yang F. (1993). Classification of apple surface features using machine vision and neural networks. Comput. Electron. Agric..

[11] Nagata M., Bato P., Mitaria M., Cao Q., Kitahara T. (2000). Study on Sorting System for Strawberry Using Machine Vision (Part 1). Jap. Soc. Agric. Mach..

[12] Behroozi N., Tavakoli T., Ghassemian H., Hadi M., Banakar A. (2013). Applied machine vision and artificial neural network for modeling and controlling of the grape drying process. Comput. Electron. Agric..

[13] Gatica G., Bestb S., Ceronic J., Lefranc G. (2013). Olive Fruits Recognition Using Neural Networks. Proc. Comput. Sci..

[14] Mancuso S., Nicese F.P. (1999). Identifying Olive (*Olea europaea*) Cultivars Using Artificial Neural Networks. Am. Soc. Hortic. Sci..

[15] Sun D. (2016). Computer Vision Technology for Food Quality Evaluation.

[16] Diaz R. (2016). Computer Vision Technology for Food Quality Evaluation.

[17] Bottle Inspection (2013). General Visions. https://www.general-vision.com/appnotes/AN_BottleInspection.pdf.

[18] Menendez A., Paillet G. (2008). Fish Inspection System Using a Parallel Neural Network Chip and the Image Knowledge Builder Application. AI Mag..

[19] Liu Y., Wei D., Zhang N. Vehicle-license-plate recognition based on neural networks. Proceedings of the IEEE on Information and Automation.

[20] Sardar S., Tewari G., Babu K.A. A hardware/software co-design model for face recognition using Cognimem Neural Network chip. In Proceeding of the IEEE on Image Information Processing.

[21] Davies M., Srinivasa N., Lin T., Chinya G., Cao Y., Choday S., Dimou G., Joshi P., Imam N., Jain S. (2018). Loihi: A Neuromorphic Manycore Processor with On-Chip Learning. IEEE Micro.

[22] Moran S., Gaonkar B., Whitehead W., Wolk A., Macyszyn L.S., Iyer S. Deep learning for medical image segmentation—Using the IBM TrueNorth neurosynaptic system. Proceedings of the SPIE Medical Imaging.

[23] Moradi S., Qiao N., Stefanini F., Indiveri G. (2018). A scalable multi-core architecture with heterogeneous memory structures for dynamic neuromorphic asynchronous processors (dynaps). IEEE Trans. Biomed. Circuits Syst..

[24] Frenkel C., Lefebvre M., Legat J., Bol D. (2019). A 0.086-mm² 12.7-pJ/SOP 64k-Synapse 256-Neuron Online-Learning Digital Spiking Neuromorphic Processor in 28-nm CMOS. IEEE Trans. Biomed. Circuits. Syst..

[25] Fried L. (2019). Making machine learning arduino compatible: A gaming handheld that runs neural networks-[Resources_Hands On]. IEEE Spectr..

[26] Lobachev I., Maleryk R., Antoschuk S., Filiahin D., Lobachev M. Integration of neural networks into smart sensor networks. Proceedings of the IEEE Xplore.

[27] Mittal S. (2019). A Survey on optimized implementation of deep learning models on the NVIDIA Jetson platform. J. Syst. Arch..

[28] Kim J., Zelinka I., Brandstetter P., Trong Dao T., Hoang Duy V., Kim S. (2019). New Neuromorphic AI NM500 and Its ADAS Application. AETA-2018 Recent Advances in Electrical Engineering and Related Sciences: Theory and Application.

[29] CogniPat SDK for Matlab (2018). General Visions. https://www.general-vision.com/download/cp_sdk_ml/.

[30] NeuroMem USB Dongle (2019). General Visions. https://www.general-vision.com/hardware/usbdongle/.

[31] López Riquelme J.A., Soto F., Suardíaz J., Sánchez P., Iborra A., Vera J.A. (2019). Wireless Sensor Networks for precision horticulture in Southern Spain. Comput. Electron. Agric..

[32] Garcia L., Parra L., Jimenez J.M., Lloret J., Lorenz P. (2020). IoT-Based Smart Irrigation Systems: An Overview on the Recent Trends on Sensors and IoT Systems for Irrigation in Precision Agriculture. Sensors.

[33] Urbano O., Perles A., Pedraza C., Rubio-Arraez S., Castelló M.L., Ortola M.D., Mercado R. (2020). Cost-Eective Implementation of a Temperature Traceability System Based on Smart RFID Tags and IoT Services. Sensors.

[34] Escolar Díaz S., Carretero Pérez J., Calderón Mateos A., Marinescu M.C., Bergua Guerra B. (2011). A novel methodology for the monitoring of the agricultural production process based on wireless sensor networks. Comput. Electron. Agric..

[35] Automated-olive-chain (2020). The internet of Food & Farm. https://www.iof2020.eu/trials/fruits/automated-olive-chain.

[36] De Jodar M., Madueño A., Lucas A., Molina J.M., Cánales A.R., Madueño J.M., Justicia M., Baena M. (2020). Deep learning in olive pitting machines by computer visión. Comput. Electron. Agric..

[37] Hecht-Nielsen R. Theory of the Backpropagation Neural Network. Proceedings of the International 1989 Joint Conference on Neural Networks.

[38] Google Coral Edge TPU (2020). Google LLC. https://coral.ai/docs/accelerator/datasheet/.

[39] Intel® Movidius™ Neural Computer Stick 2 (2020). Intel Corporation. https://www.intel.es/content/www/es/es/design/products-and-solutions/boards-kits-and-modules/movidius-neural-compute-stick-2/technical-library.html?grouping=rdc%20Content%20Types&sort=title:asc.

[40] Nvidia-Jetson-Nano (2020). Nvidia Corporation. https://www.nvidia.com/es-es/autonomous-machines/embedded-systems/jetson-nano/.

[41] TM TestNeurons SimpleScript General Visions. http://www.general-vision.com/documentation/TM_TestNeurons_SimpleScript.pdf.

[42] TM NeuroMem Technology Reference Guide (2019). General Visions. https://www.general-vision.com/documentation/TM_NeuroMem_Technology_Reference_Guide.pdf.

[43] TM_CM1K_Hardware_Manual (2017). General Visions. https://www.general-vision.com/documentation/TM_CM1K_Hardware_Manual.pdf.

[44] Halgamuge S., Poechmueller W., Glesner M. (1995). An Alternative Approach for Generation of Membership Functions and Fuzzy Rules Based on Radial and Cubic Basis Function Networks. Int. J. Approx. Reason..

[45] DS_CM1K (2014). General Visions. https://www.general-vision.com/datasheet/DS_CM1K.pdf.

[46] Neural-network The MathWorks, Inc. 1994–2017. https://es.mathworks.com/solutions/deep-learning/convolutional-neural-network.html?s_tid=srchtitle.

[47] Train Autoencoder The MathWorks, Inc. 1994–2017. http://es.mathworks.com/help/nnet/ref/trainautoencoder.html.

[48] Train Stacked Autoencoders for Image Classification The MathWorks Inc. 1994–2019. https://es.mathworks.com/help/deeplearning/examples/train-stacked-autoencoders-for-image-classification.html.

[49] Image Set Repository. https://github.com/Torras86/Olive-image-set.

[50] (2016). IDS Imaging Development Systems GmbH. https://es.ids-imaging.com/store/ui-1220se.html.

[51] QT Creator (2020). The Qt Company. https://doc.qt.io/.

[52] Dropbox (2020). Dropbox Inc. https://www.dropbox.com/developers/documentation.

[53] Braincard (2017). General Visions. https://www.general-vision.com/documentation/TM_BrainCard.pdf.

[54] NM500 Chip (2019). General Visión. https://www.general-vision.com/documentation/TM_NeuroShield_GettingStarted.pdf.

[55] I^2^C (Inter Integrated Circuit), Phillips Semiconductor, 1982. https://en.wikipedia.org/wiki/I%C2%B2C.

[56] FT232RL USB UART IC (2018). Future Technology Devices International Limited. https://www.ftdichip.com/Support/Documents/DataSheets/ICs/DS_FT232R.pdf.

[57] USB to Serial Chip CH340 (2015). SparkFun Electronics. https://cdn.sparkfun.com/datasheets/Dev/Arduino/Other/CH340DS1.PDF.

[58] Intel Curie Module (2020). Intel Corporation. https://ark.intel.com/content/www/es/es/ark/products/96282/intel-curie-module-intel-quark-se-soc.html.

[59] Teensy 4.0 USB Development Board PJRC Electronics Projects Components Available Worldwide. https://www.pjrc.com/teensy/.

[60] Google Remote Desktop (2020). Google LLC. https://support.google.com/chrome/answer/1649523?co=GENIE.Platform%3DDesktop&hl=es.

